# Reversing tozasertib resistance in glioma through inhibition of pyruvate dehydrogenase kinases

**DOI:** 10.1002/1878-0261.13025

**Published:** 2021-06-23

**Authors:** Esther P. Jane, Daniel R. Premkumar, Dhivyaa Rajasundaram, Swetha Thambireddy, Matthew C. Reslink, Sameer Agnihotri, Ian F. Pollack

**Affiliations:** ^1^ Department of Neurosurgery University of Pittsburgh School of Medicine PA USA; ^2^ Department of Neurosurgery UPMC Hillman Cancer Center PA USA; ^3^ Department of Pediatrics University of Pittsburgh School of Medicine PA USA

**Keywords:** aurora kinase, glioma, pyruvate dehydrogenase kinase, resistance, tozasertib

## Abstract

Acquired resistance to conventional chemotherapeutic agents limits their effectiveness and can cause cancer treatment to fail. Because enzymes in the aurora kinase family are vital regulators of several mitotic events, we reasoned that targeting these kinases with tozasertib, a pan‐aurora kinase inhibitor, would not only cause cytokinesis defects, but also induce cell death in high‐grade pediatric and adult glioma cell lines. We found that tozasertib induced cell cycle arrest, increased mitochondrial permeability and reactive oxygen species generation, inhibited cell growth and migration, and promoted cellular senescence and pro‐apoptotic activity. However, sustained exposure to tozasertib at clinically relevant concentrations conferred resistance, which led us to examine the mechanistic basis for the emergence of drug resistance. RNA‐sequence analysis revealed a significant upregulation of the gene encoding pyruvate dehydrogenase kinase isoenzyme 4 (PDK4), a pyruvate dehydrogenase (PDH) inhibitory kinase that plays a crucial role in the control of metabolic flexibility under various physiological conditions. Upregulation of PDK1, PDK2, PDK3, or PDK4 protein levels was positively correlated with tozasertib‐induced resistance through inhibition of PDH activity. Tozasertib‐resistant cells exhibited increased mitochondrial mass as measured by 10‐*N*‐nonyl‐Acridine Orange. Inhibition of PDK with dichloroacetate resulted in increased mitochondrial permeability and cell death in tozasertib‐resistant glioma cell lines. Based on these results, we believe that PDK is a selective target for the tozasertib resistance phenotype and should be considered for further preclinical evaluations.

## Graphical abstract

Acquired resistance to conventional chemotherapeutic agents limits their effectiveness and can cause cancer treatment to fail. Sustained exposure to tozasertib at clinically relevant concentrations conferred resistance in glioma. Tozasertib‐resistant cells exhibited increased mitochondrial mass and upregulation of pyruvate dehydrogenase kinases (PDK). Addition of PDK inhibitor resulted in increased mitochondrial permeability and cell death in tozasertib‐resistant glioma cell lines.

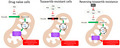

**Abbreviations**
CCCP: carbonyl cyanide m‐chlorophenyl hydrazone
DCA: dichloroacetate
DEG: differentially expressed genes
FACS: fluorescence activated cell sorting
GBM: glioblastoma
HBSS: Hank's Balanced Salt Solution
KEGG: Kyoto Encyclopedia of Genes and Genomes
NAC: acetylcysteine (*N*‐acetyl‐l‐cysteine)
NAD: nicotinamide adenine dinucleotide
PDH: pyruvate dehydrogenase
PDHC: pyruvate dehydrogenase complex
PDK: pyruvate dehydrogenase kinase
PI: propidium iodide
ROS: reactive oxygen species

## 1 Introduction

Malignant gliomas, the most common intrinsic brain tumors affecting adults and children, are aggressive, highly invasive, and neurologically destructive tumors that remain responsible for a disproportionate level of mortality among patients. In its most aggressive manifestation, glioblastoma (GBM) comprises a heterogeneous group of tumors with median survival ranges from 12 to 18 months. Despite maximum treatment efforts, survival improvements have been modest over several decades of technological advances in surgery, radiation therapy, and clinical trials of conventional and novel therapeutics. Though there is a wealth of information on basic underpinnings of glioma pathogenesis that has propelled meaningful progress in tumor subclassification, there is no satisfactory regimen available for adjuvant or salvage chemotherapy for these neoplasms [1, 2].

The aurora kinases constitute a family of serine/threonine kinases, including aurora kinases A, B, and C, whose activity is essential in mitotic spindle formation, centrosome maturation, microtubule‐kinetochore attachment, spindle checkpoint, and proper segregation of chromosomes into daughter cells [3]. Gene amplification of aurora kinase A is implicated in oncogenesis and tumor progression [4, 5].

The aurora kinase B gene coordinates accurate segregation of the chromatids at mitosis by recruiting spindle checkpoint proteins to the kinetochores which has an important role in cytokinesis [6, 7, 8, 9]. Although aurora kinase C expression is largely restricted to the testes, overexpression of aurora kinase C can recover aurora kinase B–depleted cells, suggesting some significant overlap in function [10].

Given their pivotal roles in the mitotic process during cell cycle events, aurora kinases have emerged as promising chemotherapeutic targets for cancer [11, 12]. Tozasertib (VX680), the first aurora kinase inhibitor, inhibits aurora kinases A, B, and C at low nanomolar concentrations. It inhibits cell proliferation and induces apoptosis in various human cancer cell lines [13, 14, 15, 16] including glioma [17] in preclinical and clinical settings [18]. Preclinical studies using tozasertib have shown details of its activity in various animal cancer models, validating aurora kinase as a drug target for cancer treatment [19].

Although we observed that treatment with tozasertib and other aurora kinase inhibitors increased reactive oxygen species (ROS) production and senescence and led to a significant loss in cell viability and migration, we noticed that tozasertib exposure at a dose consistent with clinically achievable plasma levels led to the appearance of a resistant population in both pediatric and adult glioma cell lines. This led us to examine the molecular mechanisms underlying the anticancer effects of tozasertib and the potential for exploitable vulnerabilities associated with acquired resistance. Using differential gene expression analysis, we identified that pyruvate dehydrogenase kinase‐4 (PDK4) mRNA and mitochondrial metabolic pathways were highly upregulated in the setting of tozasertib‐induced resistance.

The balance between the transcriptional and posttranscriptional control of the PDKs and pyruvate dehydrogenase complex (PDHC) is one way by which cancer cells alter normal pyruvate metabolism to fuel proliferation. PDHC is a major enzyme complex that regulates glucose metabolism by catalyzing oxidative decarboxylation of pyruvate to form acetyl‐coenzyme A (CoA), which enters the tricarboxylic acid (TCA) cycle and provides adenosine triphosphate (ATP) to the cell. Four isomeric forms of PDKs, namely PDK1, PDK2, PDK3, and PDK4 can phosphorylate and inactivate the PDHC, thus blocking oxidative metabolism of pyruvate by the mitochondria. PDK activity is rapidly promoted by ATP, NADH, and acetyl‐CoA, but it is inhibited by ADP, NAD^+^, CoA‐SH, and pyruvate. This process (PDK‐stimulated energy production) is shown to enhance cancer cell growth by promoting anabolic pathways [20, 21, 22]. Therefore, we explored the potency of dichloroacetate (DCA), a PDK inhibitor, that reduced mitochondrial membrane potential, and caused the release of pro‐apoptotic factors in cancer cell lines, but not normal cells [23]. In this study, we found evidence that DCA treatment contributes to mitochondrial permeabilization of tozasertib‐resistant cells, ultimately culminating in apoptosis. More broadly, this study may help in re‐orienting drug treatment strategies against aurora kinase inhibitor‐resistant tumor types by highlighting the contribution of metabolic pathways to the resistance phenotype.

## 2 Materials and methods

### 2.1 Reagents

Compounds utilized in our studies included the small molecule inhibitors tozasertib, barasertib, alisertib, ZM447439, and DCA, which were purchased from Selleck Chemicals (Houston, TX, USA). PDK4‐IN‐1 hydrochloride and VER‐246608 were purchased from MedChemExpress (Monmouth Junction, NJ, USA). Commercially available antibodies used for immunoblotting detection of aurora kinases A, B, and C, PDK1, PDK2, PDK3, PDK4, PLK1, and ẞ‐actin were purchased from Cell Signaling Technology (Danvers, MA, USA). All the fluorescent probes were purchased from Invitrogen/Molecular Probes (Eugene, OR, USA) unless otherwise stated.

### 2.2 Cell lines

We used three adult high‐grade human glioma cell lines (U87, LNZ308, and T98G), two pediatric high‐grade human glioma cell lines (KNS42 and SJG2) and SF8628, a diffuse intrinsic pontine glioma cell line (DIPG), along with normal human astrocytes. U87 and T98G cell lines were purchased from ATCC (American Type Culture Collection, Manassas, VA, USA). LNZ308 was a kind gift from N. Tribolet (Lausanne, Switzerland). The SJG2 cell line was obtained from C. Jones (Institute of Cancer Research, London, England). KNS42 was from Japanese Collection of Research Bioresources Cell Bank, Japan and SF8628, a human DIPG cell line, was purchased from Sigma‐Aldrich (St. Louis, MO, USA). Cell culture protocols of these cell lines are as described elsewhere [24]. GIBCO® Human Astrocytes were purchased from Thermo Fisher Scientific (Pittsburgh, PA, USA) and cultured using complete growth medium provided by the supplier. Cell lines used in this study did not show signs of mycoplasma contamination.

### 2.3 Generation of inhibitor‐resistant cell lines

To determine whether a pan‐aurora kinase inhibitor generates resistance, glioma cells were initially treated with a low dosage of tozasertib (10 nmol·L^−1^, approximately 10% of the physiologically relevant concentration) for 72 h. Equal amounts of vehicle (DMSO) were distributed to control cells. Viable cells were sorted using BD ARIAII cell sorter (BD Biosciences, San Jose, CA) and further maintained in complete growth medium containing double the concentration of inhibitor [24]. This process was repeated until resistance to the clinically relevant concentration (100 nmol·L^−1^) was achieved.

### 2.4 RNA sequencing, differential data analysis, and gene enrichment analysis

For RNA‐seq analysis, total RNA from triplicate samples was extracted using Qiagen RNeasy Mini Kit (Qiagen Sciences Inc., Germantown, MD, USA) as described elsewhere [24]. Paired‐end libraries (2x100 bp) were sequenced on an Illumina HiSeq3000 machine by Novogene (Sacramento, CA). These sequencing reads were further processed with STAR aligner (http://pubmed.ncbi.nlm.nih.gov/23104886/) in ‘GeneCounts’ mode, utilizing the Ensembl human transcriptome annotation (Build version GRCh38 and transcript annotation GRCh38.89). Differential expression analysis was performed using the R/Bioconductor package DESeq2, applying a false‐discovery rate threshold (FDR) of 5% and fold change of ± 1.5 [25]. Gene Set Enrichment Analysis (GSEA) was performed with the C2 and C5 BroadSets gene set collection from MsigDB3.0 [26]. Enriched Gene Ontology (GO) and Kyoto Encyclopedia of Genes and Genomes (KEGG) pathways were identified using the clusterProfiler package [27] in R and selected at the FDR threshold of 5%.

### 2.5 Western blotting analysis

After treatment with the inhibitor or vehicle, cells were harvested by trypsinization, washed in cold PBS, and lysed with cell lysis buffer (catalog number 9803; Cell Signaling Technology) containing protease inhibitor cocktail for 15 min on ice. The cell lysate was then centrifuged at 12 000 ***g*** for 15 min at 4 °C and the supernatants were isolated to quantify protein using Protein Assay Reagent (Thermo Scientific, Rockford, IL, USA). Equal amounts of protein from each treatment group were separated using SDS/PAGE and transferred onto a polyvinylidene difluoride (PVDF) membrane (Thermo Scientific). After blocking with 5% BSA, membranes were incubated with indicated antibodies overnight at 4 °C, followed by incubation with secondary antibodies for 2 h at room temperature. Immunoreactive proteins were detected with the enhanced chemiluminescence detection system (LumiGLO; Cell Signaling Technology, Catalog number 7003) as described previously [24, 28].

### 2.6 MTS cell viability assay

U87, LNZ308, T98G, KNS42, SJG2, SF8628, and normal human astrocyte cells were plated in 96‐well microtiter plates (Corning, New York, NY, USA; catalog number, 3598) at a concentration of 3 × 10^3^ cells per well in 75 μL of complete medium and incubated at 37 °C. After an overnight attachment period, an equal volume of 2X concentration inhibitor was added to the treatment groups, while the control cells received vehicle (DMSO) in equivalent volumes. In parallel, media (150 µL) without cells served as background controls. After the indicated period of incubation, cell viability was determined using a colorimetric cell proliferation assay kit (Promega, Madison, WI, USA), as reported before [24, 29].

### 2.7 Transient transfection

Logarithmically growing cells were seeded in 6‐well plates in complete growth medium and grown to 70% confluence. For transient transfection, commercially available ON‐TARGET‐plus siRNAs for human aurora kinase A (siRNA‐1, J‐003545‐26‐0002 and siRNA‐2, J‐003545‐27‐0002); aurora kinase B (siRNA‐1, J‐003326‐21‐0002 and siRNA‐2, J‐003326‐22‐0002); aurora kinase C (siRNA‐1, J‐019573‐11‐0002 and siRNA‐2, J‐019573‐13‐0002); and non‐target control siRNA (NT‐siRNA, D‐001830‐01‐05) sequences were used and transfected following the manufacturer's protocol (Dharmacon, Lafayette, CO, USA). pCMV‐6 vector (catalog number PS100001) and PDK4 (catalog number CW306843) expression plasmids obtained from Origene (Rockville, MD, USA) were used for overexpression analysis as reported previously [24, 28].

### 2.8 Transwell migration assay

Cell migration was assayed using a Boyden‐modified chamber (transwell inserts with 8‐µm pore size were precoated with Matrigel, 0.75 mg·mL^−1^; Becton Dickinson, Franklin Lakes, NJ, USA) as described previously [30, 31]. Briefly, cells were detached with trypsin/EDTA and washed twice in serum‐free growth medium by centrifugation (300 ***g***, 3 min). Cells (6 × 10^4^) in 200 μL of serum‐free growth medium were transferred to the upper chamber whereas inhibitor‐ or vehicle‐containing complete medium (500 µL) was transferred to the lower chamber. Multiple wells and multiple experiments were performed for reproducibility. Cells were fixed and stained using Diff‐Quick II staining kit (Dade Behring, Marburg, Germany) per the manufacturer's instruction after a 24‐h incubation period at 37 °C. Images were acquired under an inverted EVOS microscope (Thermo Scientific).

### 2.9 Analysis of senescence using β‐galactosidase (SA‐β‐gal) staining

Cellular senescence was determined by measurement of SA‐β‐gal activity using the Senescence Cell Staining kit (Cell Signaling Technology). Briefly, following siRNA transfection or inhibitor treatment, cells were replated (5 × 10^4^ per well) in 6‐well plates in complete medium for 24 h prior to staining. After aspirating the media, cells were washed twice with PBS and fixed using neutral buffered 4% formaldehyde for 3 min at room temperature. SA‐βgal staining solution was added and incubated at 37 °C (in an ambient condition − not in a CO_2_ incubator) for 2–4 h. After color development, cells were washed twice with PBS and images were acquired under an inverted EVOS fluorescence microscope.

### 2.10 Annexin V and propidium iodide apoptosis assay by flow cytometry

All flow cytometry experiments were performed on a BD LSR II (BD Biosciences, San Jose, CA, USA) at the Rangos Flow Cytometry Core facility at the Children's Hospital of the University of Pittsburgh. Apoptosis induction was assessed using an Annexin V, Alexa Fluor™ 488 conjugate assay kit (Thermo Scientific, Catalog number A13201; Eugene, OR, USA) as described previously [24, 29]. Cells lifted after treatment were washed with ice‐cold PBS and resuspended in binding buffer containing annexin V‐FITC and propidium iodide (PI; all provided in the kit). Cells were then incubated in a dark environment for 15 min and subsequently analyzed with flow cytometry (Becton Dickinson‐LSRII, BD Biosciences). The fluorescence activated cell sorting (FACS) histogram depicts the percentages of cells in each quadrant. Cells in the upper left quadrant (Q1, PI positive), upper right quadrant (Q2, annexin V and PI positive), lower left quadrant (Q3, annexin V and PI negative), and lower right quadrant (Q4, annexin V positive) represent dead, late apoptotic, live, and early apoptotic cells, respectively.

### 2.11 Cell cycle analysis by flow cytometry

The cell cycle profile (population of cells in sub‐G1, G1, S, G2‐M phase of cell cycle) was determined as described previously [24, 28]. Inhibitor‐treated or vehicle‐treated cells were collected and fixed with 80% ethanol on ice for 30 min. Cells were then resuspended in PBS containing 50 μg·mL^−1^ RNase and 50 μg·mL^−1^ PI before analysis using a Becton Dickinson‐LSRII flow cytometer.

### 2.12 DiOC6 labeling and detection of mitochondrial membrane depolarization by flow cytometry

Mitochondrial membrane depolarization was determined by flow cytometry as reported previously [24, 28]. Briefly, after treatment with inhibitors or vehicle control, cells were trypsinized, lifted, washed, and stained with 50 nmol·L^−1^ 3′,3′‐dihexyloxacarbo‐cyanine iodide (DiOC6; Thermo Fisher, Eugene, OR; catalog number, D273) at 37 °C for 15 min. Positively charged DiOC6 accumulates more in intact mitochondria, whereas depolarized mitochondrial membranes accumulate less DiOC6. Carbonyl cyanide 3‐chlorophenylhydrazone (CCCP, Sigma catalog number, C2759) served as a positive control. DiOC6 was excited by a broadband UV laser (488 ± 5 nm), and fluorescence emission was collected with a 525 ± 5 nm band‐pass filter.

### 2.13 Simultaneous analysis of superoxide anion ($O_{2}^{\cdot ‐}$) and hydrogen peroxide (H_2_O_2_) in living cells by flow cytometry

To simultaneously analyze superoxide anion ($O_{2}^{\cdot ‐}$) and hydrogen peroxide (H_2_O_2_), we multiplexed flow cytometry using hydroethidine (HE; Thermo Scientific, catalog number: D11347) and 2′,7′‐Dichlorodihydrofluorescein diacetate (H2DCFH‐DA; Thermo Scientific, catalog number: D399) probes as described elsewhere [32]. H2DCFH‐DA is a highly sensitive and quantifiable probe for the detection of ROS [33]. HE is oxidized rapidly to the fluorescent molecule ethidium bromide by $O_{2}^{\cdot ‐}$ [34], which occurs more slowly in the presence of O_2_ [35]. Therefore, HE is a good detector of intracellular $O_{2}^{\cdot ‐}$ [36].

At the end of the incubation period with inhibitors or vehicle control, cells were harvested by trypsinization, washed twice with prewarmed PBS, and pelleted by centrifugation at 300 ***g*** for 5 min. After aspirating PBS, cells were resuspended in 1 mL prewarmed complete medium containing 1 µm HE (freshly prepared from 10 mm stock dissolved in DMF, *N*,*N*‐Dimethylformamide, Sigma, catalog number: 227056) and incubated in the dark at 37 °C for 30 min. After incubation, cells were pelleted, washed, resuspended in PBS, and incubated with 2 µm H2DCFH‐DA (freshly prepared from 10 mm stock dissolved in DMSO) in the dark for 30 min at 37 °C. Then, cells were washed and resuspended in PBS and measured in a flow cytometer. The dyes (H2DCFH‐DA and HE) used in this protocol were excited by the blue laser (Becton Dickinson flow cytometer, solid state blue laser, 488 nm), and emissions were detected through 520/20, 575/25 nm filters, respectively.

### 2.14 Measurement of mitochondrial mass by flow cytometry

The mitochondrial mass was measured using 10‐*N*‐nonyl‐acridine orange (NAO; Thermo Scientific, catalog number: A1372), a fluorescent dye that binds with cardiolipin (CL), a phospholipid found in the inner membrane of mitochondria [37]. After treatment with inhibitors, cells were harvested by trypsinization and processed for flow cytometric analysis as described previously [24, 38]. NAO was excited by a broadband UV laser (488 ± 5 nm), and fluorescence emission was collected with a 525 ± 5 nm band‐pass filter.

Typically, 10 to 30 000 cells were analyzed for each sample. We prepared blanks (unstained samples), stained samples (with fluorochrome), and samples stained with fluorochrome with appropriate positive and negative controls to ensure all the experimental conditions were working properly. The mean fluorescence value was expressed after subtracting autofluorescence of control cells at the same concentration of the fluorescent probe.

### 2.15 Pyruvate dehydrogenase activity assay

Pyruvate dehydrogenase (PDH) activity was assessed using the PDH Activity Colorimetric Assay Kit from BioVision (Milpitas, CA, USA; Catalog number K679‐100). After overnight incubation at 37 °C, the cells were treated with inhibitor or an equal amount of vehicle (DMSO). Cells were collected by centrifugation, washed, and lysed to measure the PDH activity. Cells were lysed with lysis buffer (provided in the kit), and protein concentration was determined using BCA Protein Assay Kit with BSA standards according to the manufacturer's protocol (Thermo Scientific, Rockford, IL, USA; Catalog number 23228). Equal quantities of protein were taken from each sample, and the reaction volume was adjusted to 50 μL with PDH Assay Buffer and transferred to a 96‐well microtiter plate (Thermo Scientific, Pittsburgh, PA, USA; catalog number, 12565501). Triplicate assays were performed on each sample with appropriate positive and negative controls provided by the manufacturer. The plate was incubated at 37 °C in the dark, and absorbance was measured using a HTX Multimode Plate Reader (BioTek Instruments Inc., Model number S1FA; Winooski, VT, USA) at 450 nm for a 30 min interval. An NADH standard curve was prepared in parallel, and the slopes of the kinetic measurements were used to determine a rate of NADH produced per minute per microgram (PDH activity) of protein as described in the protocol.

### 2.16 Statistical analysis

The data are expressed as mean ± SD. Statistical analyses were conducted using Prism 6.0 graphpad (San Diego, CA, USA) software. To identify differences among subgroups, ANOVA analysis was conducted for multigroup comparisons followed by *post hoc* Tukey's test. Direct comparisons were conducted using an unpaired two‐tailed Student's *t*‐test.

## 3 Results

### 3.1 Aurora kinase is essential for glioma cellular proliferation

**Fig. 1:**
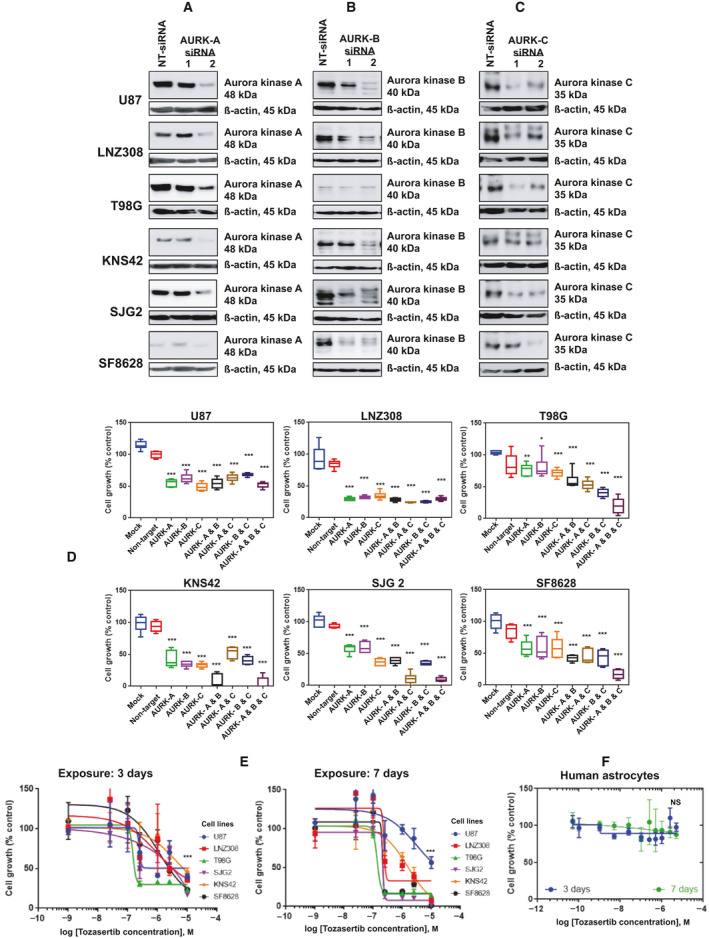
Aurora kinase is essential for glioma cellular proliferation. (A–C) Logarithmically growing adult (U87, LNZ308, and T98G) and pediatric (KNS42 and SJG2) high‐grade glioma cell lines and SF8628, a diffuse intrinsic pontine glioma cell line were either transfected with non‐target siRNA (NT siRNA) or two aurora kinase A, B, or C siRNAs. Twenty micrograms of protein were loaded on a SDS/PAGE. Aurora kinase A, B, and C and ẞ‐actin expression were evaluated by western blotting. These experiments were performed at least three times, and a representative blot is presented. (D) Cells were either mock transfected (with no siRNA) or transfected with non‐target siRNA (NT siRNA) or aurora kinase A, B, or C siRNA, alone or in combination. Seventy‐two‐hour post‐transfection, cell growth was assessed by MTS assay. Absorbance was recorded at 490 nm using a Synergy HTX multimode microplate reader. The values (*n* = 4 wells per condition) are represented as mean ± standard deviation of three separate experiments. Results were analyzed by the Tukey *post hoc* ANOVA (*P < 0.02, **P < 0.005, and ***P < 0.0001 compared with non‐target siRNA‐transfected group). Malignant human glioma cells (E) or non‐neoplastic human astrocytes (F) (3 × 10^3^) were seeded on 96‐well plates. After overnight attachment period, cells were treated with the pan‐aurora kinase inhibitor tozasertib for 3 days or 7 days. Control cells received vehicle (DMSO). Cell growth was assessed by spectrophotometric measurement of MTS bioreduction. Graphs illustrating the relationship between tozasertib vs glioma (E) or non‐neoplastic human astrocyte (F) cell growth. Points (*n* = 4 wells per condition) represent the mean of three measurements ± standard deviation. Results were analyzed from three independent experiments (NS, not significant; ***P < 0.0001; DMSO‐treated control vs tozasertib‐treated cells; unpaired two‐tailed *t*‐test).

Multiple investigators have shown that the Aurora protein kinase family (consisting of aurora kinase A, kinase B, and kinase C) controls several aspects of cell division in mammalian cells [3]. To examine the effect of the aurora kinases in glioma cells, two siRNAs specific to aurora kinase A, aurora kinase B, aurora kinase C, and a negative control siRNA (non‐target siRNA) were transiently transfected into adult (U87, LNZ308, T98G) and pediatric (KNS42, SJG2, and SF8628) cell lines. After 72‐h post‐transfection, cells were lysed and subjected to western blot analysis (Fig. 1A, aurora kinase A silencing; Fig. 1B, aurora kinase B silencing; Fig. 1C, aurora kinase C silencing). More than 60% of endogenous aurora kinases was suppressed by addition of aurora kinase siRNA‐2 (hence siRNA‐2 was used for all the subsequent studies). In contrast, a nonspecific siRNA was shown to have no substantial effect on endogenous aurora kinase expression. Because of striking morphological changes (data not shown) in glioma cells depleted of either aurora kinase A, aurora kinase B, or aurora kinase C, we investigated the function *in vitro*. First, cell growth was determined by MTS assay. Genetic silencing of aurora kinase A or aurora kinase B or aurora kinase C inhibited cell growth significantly compared to non‐target siRNA‐transfected cells or mock‐transfected control cells. Although silencing aurora kinases (individually) inhibited cell growth, co‐transfecting all three aurora kinase siRNAs significantly suppressed cell growth (48%, 85%, 84%, 91%, 91%, and 93% in U87, LNZ308, T98G, KNS42, SJG 2, and SF8628 cells, respectively), suggesting that silencing multiple aurora kinases produced more growth inhibition and that glioma cells require the combined activities of all three aurora kinases for survival and proliferation (Fig. 1D).

These observations prompted us to test tozasertib (VX‐680, MK‐0457), a pan‐aurora kinase inhibitor, to assess the efficacy of pharmacological inhibition of aurora kinases on growth in pediatric and adult glioma cell lines. When we examined the effect of tozasertib on cell proliferation, we found a dose‐ and time‐dependent inhibition in all glioma cell lines (Fig. 1E). To examine whether tozasertib had toxicity toward non‐neoplastic cells, human astrocytes were treated with increasing inhibitor concentrations and cell viability was quantitated by MTS assay after 3 and 7 days. As shown in Fig. 1F, as high as 5 µmol·L^−1^ resulted in no measured toxicity, suggesting that tozasertib acts selectively against glioma cells, sparing non‐neoplastic astrocytes.

### 3.2 Inhibition of aurora kinases induces G2M arrest and endoreduplication in human glioma cell lines

After establishing that the aurora kinases are essential for glioma cell proliferation, we employed both RNA interference and a pharmacological inhibition approach to test the effect on cell cycle profile by flow cytometry. Interestingly, we noticed that silencing aurora kinase A, B, or C had a distinctive effect on cell cycle distribution sizes compared to non‐transfected siRNA (Fig. S1A,B).

**Fig. 2:**
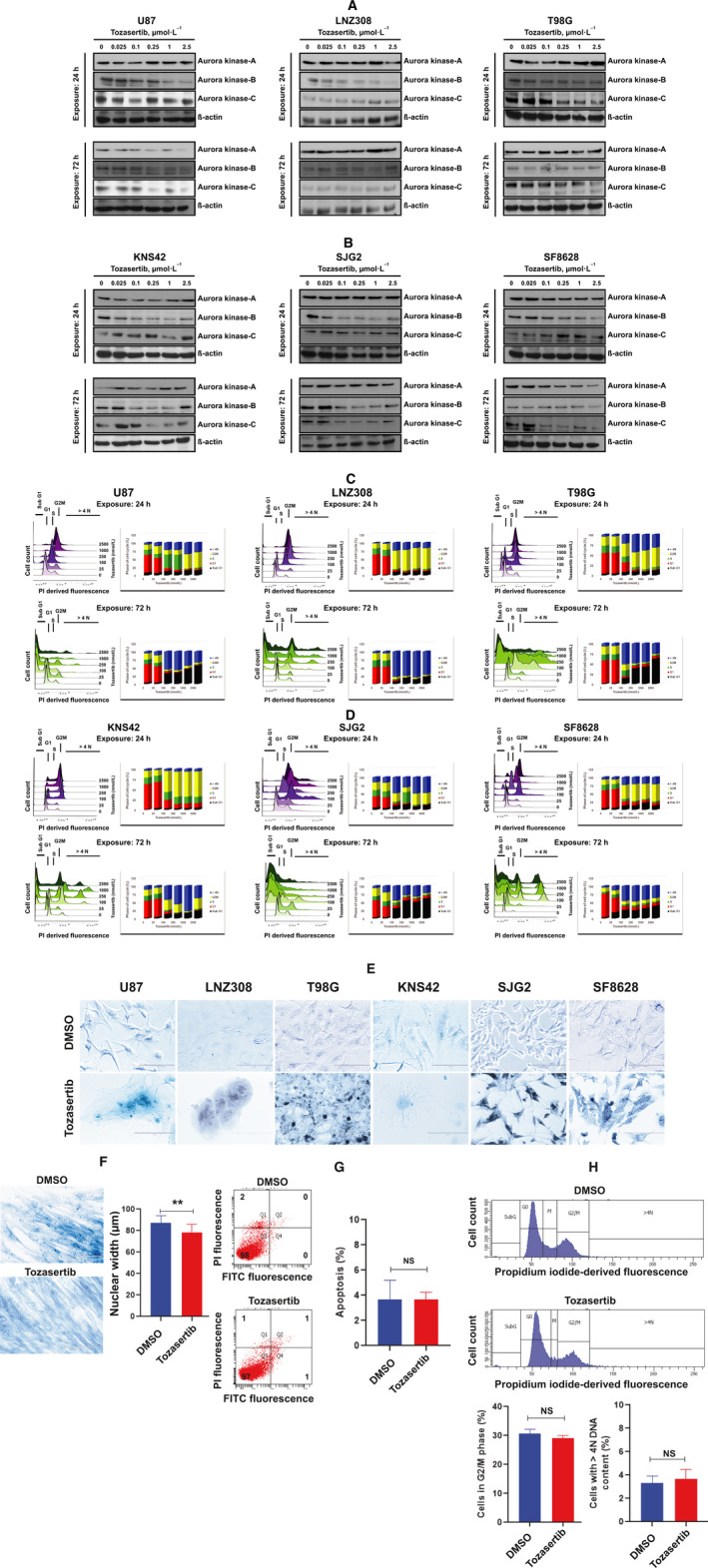
Tozasertib induces G2M arrest, endoreduplication, and apoptosis in human glioma cell lines. U87, LNZ308, and T98G (A) or KNS42, SJG2, or SF8628 (B) cells were treated with tozasertib for 24 h (upper panel) or 72 h (lower panel). Equivalent concentrations of vehicle (DMSO) served as control (0). After treatment, the cells were washed with PBS and protein was extracted. Twenty micrograms protein was separated on a SDS/PAGE. Aurora kinase A, B, and C and ẞ‐actin expression were evaluated by western blotting analysis. These experiments were performed at least three times, and a representative blot is presented. (C, D) Adult (U87, LNZ308, and T98G) and pediatric (KNS42, SJG2, and SF8628) cells were exposed to tozasertib for 24 h (upper panel) or 72 h (lower panel), and cell cycle analysis was performed. After treatment, the cells were trypsinized, lifted, washed, and stained with PI. Fluorescence of the PI‐stained cells was measured using a Becton Dickinson flow cytometer. The data in the left panel represent the cellular DNA content frequency histograms from a representative study and the percentage of cells with fractional DNA content of cells in sub‐G1, G1, S, G2M, or more than 4N DNA content from three additional experiments of the cycle were presented in the right panel. When cells were treated with tozasertib (100 nmol·L^−1^ or higher) for 24 h, a large population of cells accumulated in the G2M phase of the cell cycle (note the yellow shaded bar in the upper panels). However, with 72‐h exposures, cells accumulated to a greater extent in 4N (note the blue‐shaded bar in the lower panels) or higher apoptotic cell populations (black‐shaded bar in the lower panels). (E) The cell (U87, LNZ308, T98G, KNS42, SJG2, and SF8628) morphology of vehicle control (DMSO) and tozasertib‐treated cells (100 nmol·L^−1^ for 72 h). Bars represent 100 µm. (F) Non‐neoplastic human astrocytes were incubated with tozasertib (100 nmol·L^−1^) or DMSO for 72 h. Cell morphology (left panel; bar represents 100 µm) and nuclear width were evaluated (right panel. *n* = 200). Results were analyzed from three independent experiments (**P < 0.003; DMSO‐treated vs tozasertib‐treated cells; unpaired two‐tailed *t*‐test). (G) Human astrocytes (seeded at a density of 10^5^ cells per well in a six‐well plate) were treated with tozasertib (100 nmol·L^−1^) or DMSO for 72 h. Apoptosis was assessed by annexin V‐FITC and PI staining and FACS analysis (representative FACS histogram is shown in the left panel and the quantitative results in the right panel). All data are representative of three independent experiments (NS, not significant; unpaired two‐tailed *t*‐test). (H) Human astrocytes (10^5^ per well in a six‐well plate) were seeded and treated with tozasertib (100 nmol·L^−1^) or DMSO for 72 h. Cell cycle profile (upper panel) was assessed by FACS analysis of cells labeled with PI (*X*, axis). Tozasertib does not induce accumulation of cells in the G2M phase of the cell cycle (representative FACS histogram in the top and middle panel) or increase the number of cells within the G2M phase of the cell cycle or cells with > 4N DNA content (quantitative data at the bottom panel). The values are represented as mean ± standard deviation of three separate experiments (NS, not significant; unpaired two‐tailed *t*‐test).

After examining the effect of inhibiting individual aurora kinases by genetic silencing, we next used tozasertib, the pharmacological pan‐aurora kinase inhibitor, to see how glioma cells responded when deregulating all three aurora kinases. Adult (Fig. 2A) and pediatric (Fig. 2B) glioma cells were treated with varying concentrations of tozasertib for 24 h or 72 h. Western blot analysis showed that tozasertib modestly inhibited aurora kinase protein expression levels in a dose‐ and time‐dependent manner (Fig. 2A,B). Next, we examined the cell cycle profile changes upon tozasertib treatment. Interestingly, the 24‐h cell cycle profile was markedly different from 72 h. Twenty‐four‐hour treatment led to the accumulation of cells in G2M phase in a concentration‐dependent manner (Fig. 2C upper panel for adult high‐grade glioma cell lines and Fig. 2D upper panel for pediatric high‐grade glioma cell lines). Long‐term exposure (72 h) to tozasertib significantly decreased the number of cells in the G2M phase of the cell cycle (Fig. 2C lower panel for adult high‐grade glioma cell lines and Fig. 2D lower panel for pediatric high‐grade glioma cell lines). As shown in Fig. 2C,D, there was a significant increase in the number of cells (in all six cell lines) in G2M phase after treatment with tozasertib after 24 h. The percentage of U87, LNZ308, T98G, KNS42, SJG2, and SF8628 cells in G2M phase was increased from 15, 16, 22, 17, 24, and 16%, respectively, in DMSO‐treated controls to 47%, 63%, 47%, 62%, 33%, and 52% after 24‐h treatment with 2.5 μmol·L^−1^ tozasertib, but declined to 4%, 3%, 2%, 33%, 4%, and 10% after 72‐h treatment with tozasertib (Fig. S1C). After 72‐h treatment, we noticed a significant increase in the percentage of cells having more than 4N DNA content as evidenced by the appearance of a population readily shifted to right. Tozasertib also produced a concentration‐ and time‐dependent increase in the number of cells in the sub‐G1 fraction of cell cycle (Fig. 2C,D), a portion of dead cells, compared to cells incubated with vehicle control (Fig. S1C). Taken together, the results suggest that tozasertib induces a notable cell cycle arrest and induces apoptosis in adult and pediatric glioma cells, contributing to its anticancer effect.

After assessing the accumulation of cells with > 4N DNA content (by flow cytometry‐cell cycle profile), we examined the nuclear morphology by visualizing whether there was any increase in nuclear size (due to endoreduplication) by fluorescence microscopy. Cells treated with 100 nmol·L^−1^ tozasertib for 72 h showed a substantial increase in nuclear width (Fig. 2E). The percentage of U87, LNZ308, T98G, KNS42, SJG2, and SF8628 cells demonstrating > 4N DNA content was increased from 6%, 5%, 8%, 12%, 7%, and 5%, respectively, in DMSO‐treated control to 56%, 78%, 52%, 43%, 45%, and 46.5%, respectively, in 100 nmol·L^−1^ tozasertib after 72‐h treatment (Fig. S1C). These results (observed in adult and pediatric glioma cell lines) prompted us to compare the effect in normal human astrocytes. We measured cell cycle profile and nuclear characteristics of cells undergoing apoptosis and found out that unlike glioma cells, tozasertib treatment did not affect human astrocyte cell morphology and nuclear size (Fig. 2F), viability (Fig. 2G), and cell cycle profile (Fig. 2H), suggesting that tozasertib exhibits selective effect on neoplastic vs non‐neoplastic astrocytes.

### 3.3 Suppression of aurora kinases induces cellular senescence and inhibits migration

**Fig. 3:**
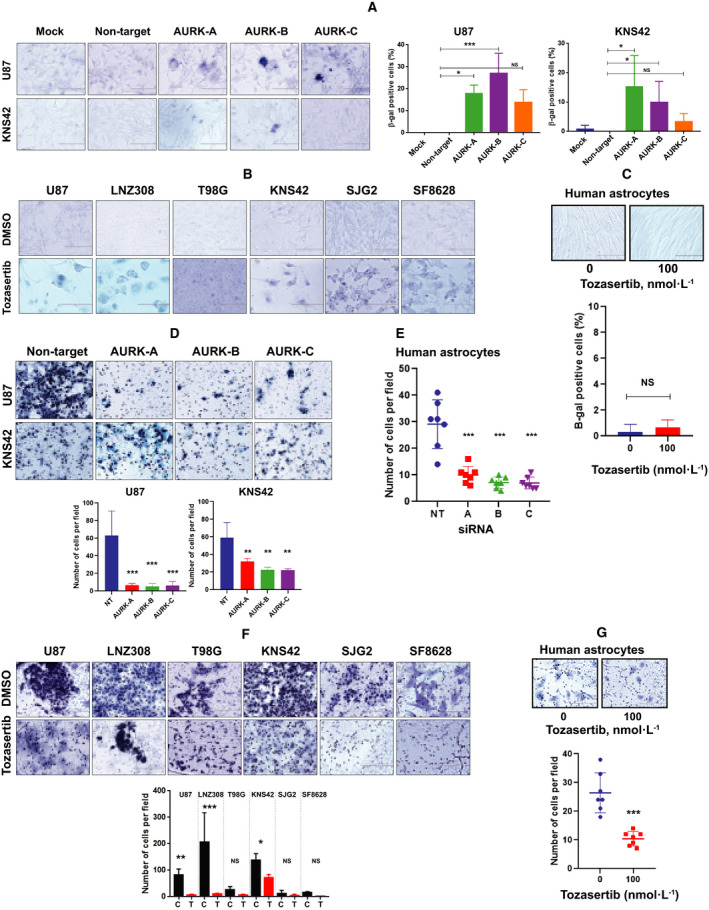
Suppression of aurora kinases induces cellular senescence and inhibits migration. (A) U87 and KNS42 cell types were either mock‐transfected with no siRNA or a non‐target RNA or transfected with siRNA that was directed against aurora kinase A, B, or C. Three days after transfection, cells were stained for SA‐β‐gal activity, a marker for cellular senescence. Morphology of cells staining positive for SA‐β‐gal is shown in the left panel. Bar represents, 100 µm. These experiments were performed at least three times, and the values are represented as mean ± standard deviation. Results were analyzed for statistical significance by Tukey's ANOVA test (*P < 0.05; ***P < 0.0001; NS, not significant). (B) Adult (U87, LNZ308, and T98G) and pediatric (KNS42, SJG2, and SF8628) glioma cells were treated with or without tozasertib (100 nmol·L^−1^). Control cells received DMSO. Three days after treatment, cells were stained for SA‐β‐gal activity. Morphology of cells staining positive for SA‐β‐gal is shown. The results are expressed as the mean ± SD from three independent experiments. Scale bar, 100 µm. (C) Non‐neoplastic human astrocytes were treated with or without tozasertib (100 nmol·L^−1^). Control cells (0) received DMSO. Three days after treatment, cells were stained for SA‐β‐gal activity. Morphology of cells staining positive for SA‐β‐gal is shown in the upper panel (scale bar = 100 µm). The results are expressed as the mean ± SD from three independent experiments. Statistical analysis was carried out with an unpaired two‐tailed *t*‐test (NS, not significant). (D) U87 and KNS42 cell types were either transfected with a non‐target RNA or transfected with siRNA that was directed against aurora kinase A, B, or C. Three days after transfection, cell migration was assessed. Representative digital images taken from one experiment are presented (scale bar = 100 µm). The results are expressed as the mean ± SD from three independent experiments. Statistical analysis was carried out with Tukey's ANOVA (**P < 0.005 and ***P < 0.0001 compared with non‐target siRNA‐transfected group). (E) Non‐neoplastic human astrocytes were either transfected with a non‐target siRNA (NT) or transfected with siRNA that was directed against aurora kinase A, B, or C. Three days after transfection, cell migration was assessed. Data are the mean ± SD of three independent experiments. Statistical significance was assessed by Tukey's ANOVA test (***P < 0.0001 compared with non‐target siRNA‐transfected group). (F) U87, LNZ308, T98G, KNS42, SJG2, and SF8628 cells were treated with 100 nmol·L^−1^ tozasertib (T). Control (C) cells received DMSO. Three days after treatment, cell migration was assessed (upper panel). Bar represents, 100 µm. All data (bottom panel) are representative as mean ± standard deviation of three separate experiments (*P < 0.05, **P < 0.005, ***P < 0.0001, unpaired two‐tailed *t*‐test). (G) Non‐neoplastic human astrocytes were treated with 100 nmol·L^−1^ tozasertib. Control (0) cells received DMSO. Three days after treatment, cell migration was assessed (upper panel). Bar represents, 100 µm. The values (bottom panel) are represented as mean ± standard deviation of three separate experiments (***P < 0.0001). Statistical significance was assessed with an unpaired two‐tailed *t*‐test.

Because cellular senescence reflects the process of cell cycle arrest that is accompanied by the exhaustion of replicative potential [39], we determined whether genetic silencing or pharmacological inhibition of aurora kinases were capable of influencing glioma cell senescence. Cells were transfected with either aurora kinase siRNA or non‐target siRNA and cellular senescence was assessed as described in the Methods. Silencing aurora kinases significantly resulted in large, flat, vacuolated cells which stained positive for SA‐β‐gal, a marker for senescence (Fig. 3A). Similar results were found with tozasertib treatment (Fig. 3B), suggesting that inhibiting aurora kinases contributes to glioma cell senescence. In an attempt to examine the cellular senescence in human astrocytes, cells were grown in the presence or absence of tozasertib (100 nmol·L^−1^), and SA‐β‐gal staining was performed after 72 h. Surprisingly, unlike adult and pediatric glioma cell lines, treatment with tozasertib did not induce cellular senescence in human astrocytes (Fig. 3C).

To determine the functional relevance of aurora kinases in glioma cell migration, an RNA interference and pharmacological approach was taken. Cells were transfected with aurora kinase A, B, or C siRNA or non‐target siRNA. After introduction of aurora kinase siRNA in U87 cells (an adult glioma cell line), we noticed a significant reduction in migration compared to cells transfected with nonspecific siRNA. In parallel, we also examined these same siRNA experiments in a pediatric glioma cell line (KNS42), and obtained similar observations, suggesting disruption of endogenous aurora kinases resulted in suppression of glioma cell migration (Fig. 3D). We extended this observation in human astrocytes. As shown in Fig. 3E, silencing aurora kinase A or B or C caused a significant reduction in human astrocyte cellular migration. Furthermore, pharmacological inhibition of aurora kinases produced a similar effect in both glioma cell lines (Fig. 3F) and normal human astrocytes (Fig. 3G). We noticed LNZ308 cells were most invasive than all other cell lines. Of note, although most glioma cells appear to have a migratory phenotype, it has been shown that they rarely metastasize outside the cranium [40]. In contrast to U87, LNZ308, and KNS42, we noticed a poor migratory potential in T98G, SJG2, and SF8628 cell lines, which may reflect the heterogeneity commonly noted in these tumors.

### 3.4 Tozasertib induces superoxide anion and reactive oxygen species generation

**Fig. 4:**
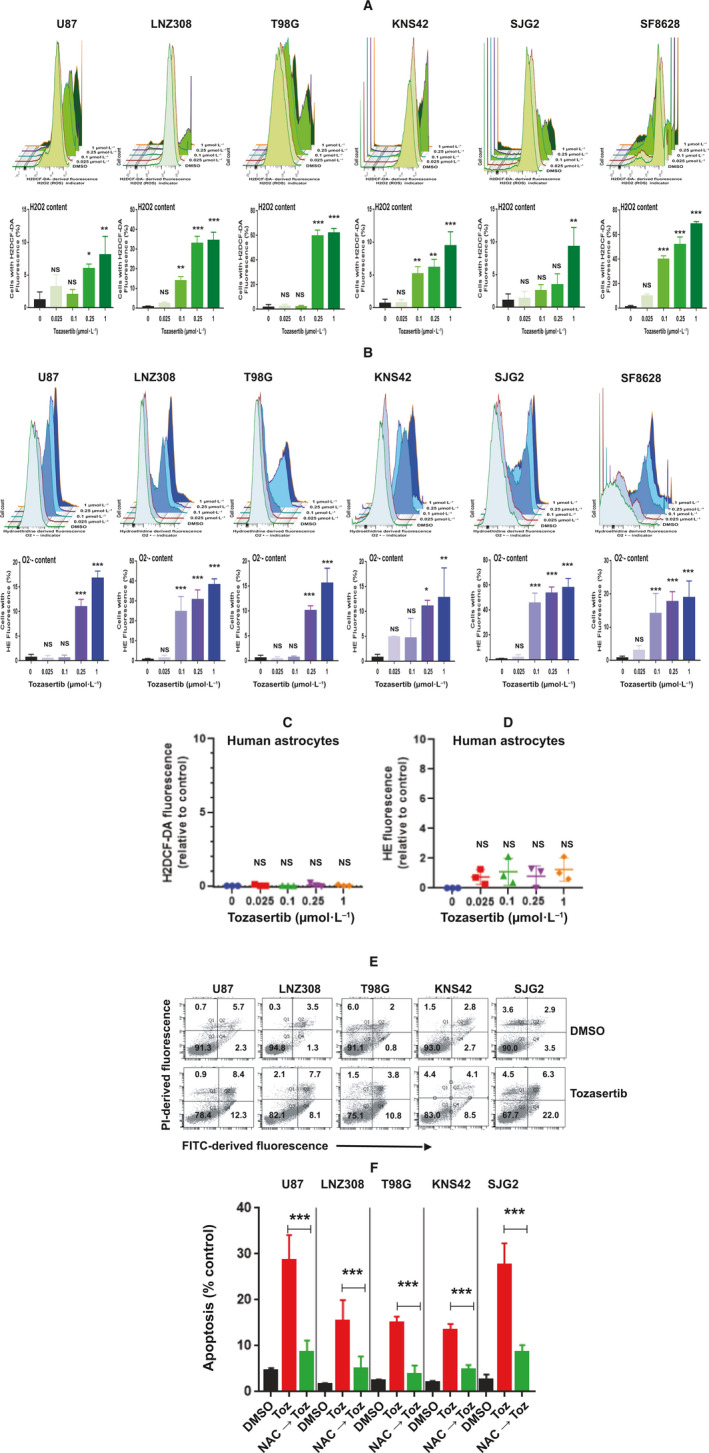
Tozasertib induces superoxide anion and ROS generation. (A, B) Adult (U87, LNZ308, and T98G) and pediatric (KNS42, SJG2, and SF8628) glioma cell lines were treated with the indicated concentrations of tozasertib for 72 h, and then, the cells were labeled with H2DCFDA and hydroethidine to analyze hydrogen peroxide (A) and superoxide anion (B), respectively, by flow cytometry. All experiments were performed at least three times. The representative FACS histogram is shown in the upper panel. Treatment with tozasertib was accompanied by an increase of H2DCF‐DA fluorescence (shift to the right in a dose‐dependent manner; A, upper panel) and HE fluorescence (shift to the right in a dose‐dependent manner; B, upper panel), which is proportional to the intracellular H_2_O_2_ and $O_{2}^{\cdot ‐}$ content, respectively. Quantitative data from three separate experiments are presented in the lower panel (A, H_2_O_2_ content and B, $O_{2}^{\cdot ‐}$ content). The values are represented as mean ± standard deviation of three separate experiments. Statistical significance was assessed with Tukey's ANOVA (NS, not significant; *P < 0.01, **P < 0.005, and ***P < 0.0001 compared with DMSO‐treated control). (C, D) Non‐neoplastic human astrocytes were treated with the indicated concentrations of tozasertib for 72 h, and then, the cells were labeled with H2DCFDA and hydroethidine to analyze H_2_O_2_ and $O_{2}^{\cdot ‐}$ content, respectively. Quantitative data from three independent experiments are presented here (C, H_2_O_2_ content and D, $O_{2}^{\cdot ‐}$ content). Statistical analysis was carried out with Tukey's ANOVA (NS, not significant). (E) Cells were treated with 100 nmol·L^−1^ tozasertib for 72 h. Vehicle‐treated (DMSO) cells served as control. Apoptosis was assessed by Annexin V‐FITC and PI staining and flow cytometric analysis. Annexin V is plotted on the x‐axis, and PI is plotted on the y‐axis. Cells in the lower left quadrant represent live cells; cells in the lower right quadrant (annexin V positive) reflect number of cells in the early apoptotic phase; cells in the upper right quadrant (annexin V/PI positive) reflect the number of cells in the late apoptotic phase; cells in the upper left quadrant (PI positive) represent dead cells. These experiments were performed at least three times, and a representative annexin histogram is presented. (F) Cells were treated with tozasertib in the presence or absence of NAC (0.25 mmol·L^−1^). After 72 h, cells were stained with annexin V and PI and quantitative measurements of apoptosis was performed by flow cytometry. Graph represents the percentages of apoptotic cells acquired from three independent experiments for each cell type (mean ± SD). Statistical significance was assessed with Tukey's ANOVA (***P < 0.0001).

Aurora kinase inhibition has been shown to alter various antioxidant enzymes such as superoxide dismutase and catalase, which are important to maintain proper cellular redox states [41, 42]. The impact of mitochondrial activities on cellular physiology is not only restricted to ATP production, but also the generation of ROS [43]. Although aurora kinase inhibitors lead to excessive ROS and increased cell death [44, 45, 46], information regarding tozasertib on glioma has been limited. First, we analyzed whether tozasertib treatment could result in increased hydrogen peroxide (H_2_O_2_) and superoxide anion ($O_{2}^{\cdot ‐}$). As shown in Fig. 4A, both adult and pediatric glioma cells accumulated significant intracellular levels of H_2_O_2_ after tozasertib treatment compared with DMSO‐treated cells in a dose‐dependent manner, as determined with the cell permeable ROS indicator H2‐DCFDA. Similarly, tozasertib treatment significantly increased $O_{2}^{\cdot ‐}$ levels (Fig. 4B). In contrast, tozasertib‐induced oxidative stress was undetectable in non‐neoplastic human astrocytes (Fig. 4C,D). We next assessed cell death after tozasertib treatment. FITC‐labeled annexin V and PI staining followed by flow cytometry analysis revealed a significant change in the cell survival. For instance, 72‐h treatment with 100 nmol·L^−1^ tozasertib significantly decreased cell survival (Fig. 4E), whereas pretreating cells with the ROS scavenger [acetylcysteine (*N*‐acetyl‐l‐cysteine) (NAC)] significantly reduced cell death (Fig. 4F), suggesting that oxidative stress is indeed a major consequence of tozasertib‐induced cell death in malignant glioma cell lines.

### 3.5 Sustained inhibition of aurora kinases leads to acquired resistance in glioma

**Fig. 5:**
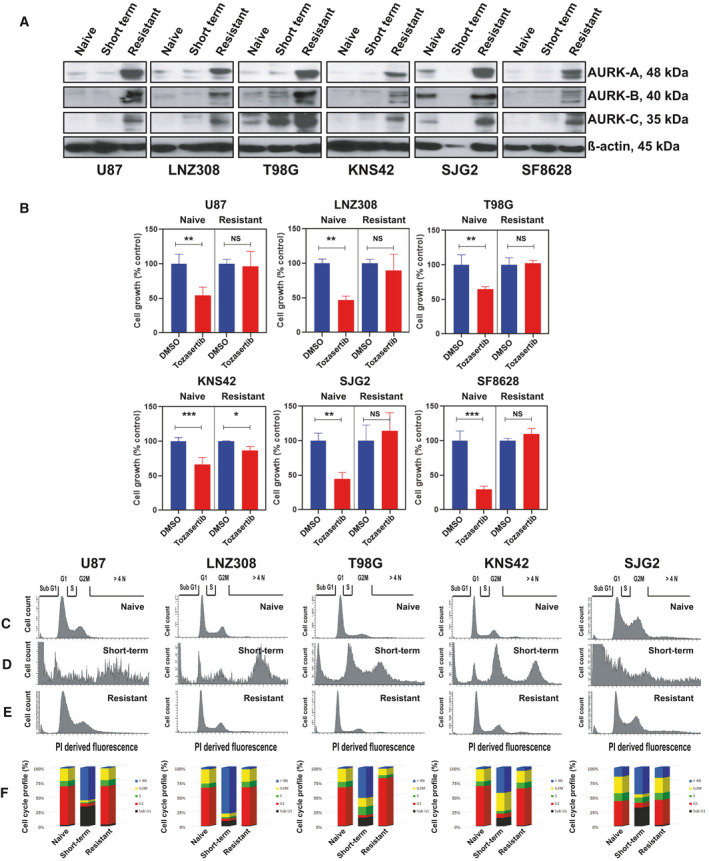
Sustained inhibition of aurora kinases leads to acquired resistance in glioma. (A) Whole cell extracts from drug‐naïve cells or those treated with tozasertib for 72 h (short‐term), or tozasertib‐resistant cells were subjected to western blot analysis. Expression of β‐actin served as loading control. Two additional experiments produced similar results. (B) Drug naïve or tozasertib‐resistant (resistant) cells were allowed to attach overnight in a 96‐well plate and treated with tozasertib for 72 h. Cell viability was assessed by MTS assay. The data represent the mean ± SD of three three independent experiments (DMSO‐treated vs tozasertib‐treated cells; unpaired two‐tailed *t*‐test. NS, not significant; *P < 0.05, **P < 0.005, ***P < 0.0001). (C–F) Drug naïve or tozasertib‐resistant (resistant) cells were allowed to attach overnight in a six‐well plate. Drug‐naïve cells were either treated with tozasertib for 72 h (short‐term) or with an equal amount of vehicle (DMSO). After treatment, the cells were trypsinized, lifted, washed, and stained with PI. Fluorescence of the PI‐stained cells was measured using Becton Dickinson flow cytometer. (C–E) Represent the cellular DNA content frequency histograms from a representative study and the average estimate of percentage of cells with fractional DNA content in sub‐G1, G1, S, G2M, or > 4N DNA content from 3 additional experiments of the cycle were presented in (F) When drug‐naïve cells were treated with the inhibitor for 72 h, a large population of cells accumulated in the sub‐G1 (pro‐apoptotic phase of the cell cycle representing cells undergoing apoptosis/necrosis. Also note the black‐shaded box in F) or cells containing > 4N DNA content (compare Fig. 5C vs Fig. 5D. Also note the blue‐shaded box in F). However, this effect was not seen in the resistant cells (compare Fig. 5C vs Fig. 5E), suggesting that the sustained exposure to inhibition led to the selection of a population of cells that cycle normally at a concentration of inhibitor that induces cell cycle arrest and apoptosis in drug‐naïve counterparts. Quantitative measurements of cell cycle profile were performed, and the percentage of cells accumulated in different phases of the cell cycle from three different experiments are presented in (F).

Although clinically relevant concentrations of tozasertib inhibited cell growth and migration, and induced senescence in a high percentage of glioma cells, sustained exposure consistently yielded a subpopulation of cells that escaped inhibition and retained the ability to proliferate like drug‐naïve control cells. To investigate how long‐term inhibition of aurora kinase could lead to acquired drug resistance, a panel of both adult and pediatric glioma cell lines were chronically treated with physiologically relevant concentrations of tozasertib for several weeks (Fig. S2). Morphological analysis revealed that there were no discernable differences between drug‐naïve and resistant cells (data not shown). Next, we analyzed the expression levels of the aurora kinase A, B, and C proteins. Interestingly, these three protein's expression levels were significantly increased in the tozasertib‐resistant cell population compared to drug‐naïve controls or short‐term treated cell lines (Fig. 5A). When we examined the effect of tozasertib on cell proliferation, as shown earlier, tozasertib inhibited cell growth in drugnaïve cells (Fig. 5B). However, we did not observe any discernable change in cell viability between the control cells and the long‐term inhibitor‐treated resistant glioma cells (Fig. 5B). Cell cycle analysis clearly demonstrated that short‐term exposure (100 nmol·L^−1^ tozasertib for 72 h) led to a substantial change in the cell cycle profile compared to untreated controls (compare Fig. 5C vs Fig. 5D). In contrast, no visible change was evident in the cell cycle profile between drug‐naïve cells vs tozasertib‐resistant cells (compare Fig. 5C vs Fig. 5E). Consistent with our observation in Fig. 2C, treatment with 100 nmol·L^−1^ tozasertib for 72 h induced the accumulation of cells with > 4N or greater DNA content, which were absent from resistant cells (Fig. 5F). Assessed quantitatively from multiple experiments (Fig. S3A), we observed a significant difference in the population of cells accumulated in G1, S, and G2M phase of cell cycle content between short‐term treated vs resistant cells. Furthermore, in short‐term treatment (100 nmol·L^−1^ for 72 h), cell death occurred to a significantly greater extent than in resistant cells (sustained exposure to tozasertib in 100 nmol·L^−1^), as shown by the appearance of a sub‐G1 peak in the DNA content analysis by flow cytometry (Fig. 5F and Fig. S3A). Together these results suggest the possibility that, in glioma cell lines, sustained exposure to tozasertib at a clinically relevant concentration appears to promote a high degree of resistance.

Then, to examine whether cells chronically treated with tozasertib exhibited cross‐resistance to other specific aurora kinase inhibitors, parental and tozasertib‐resistant cells were treated with alisertib, barasertib or ZM447439. Treatment of control cells with alisertib, barasertib, and ZM447439 notably reduced viability. However, tozasertib‐resistant cells showed a minimal effect (Fig. S3B), suggesting that chronic treatment with an aurora kinase inhibitor can lead to development of cross‐resistance to other aurora kinase inhibitors in glioma cell lines that were initially highly sensitive to these agents. Because multiple studies have shown that the survival advantage of certain cancers depends on the activity of overexpressed aurora kinases and polo‐like kinases (PLK) [47, 48] and both kinases co‐regulate many cellular processes governing cell division by activating specific cyclin‐dependent kinases (CDKs) and their associated cyclins, we examined the expression of PLK1 protein in naïve, short‐term tozasertib‐treated, and tozasertib‐resistant adult and pediatric glioma cell lines. As shown in Fig. S3C, PLK1 protein expression level is high in the logarithmically growing adult (U87 and LNZ308) and pediatric (SJG2 and SF8628) glioma cell lines. However, short‐term treatment with tozasertib significantly inhibited PLK1 protein expression, while all the tozasertib‐resistant cell lines (sustained exposure to tozasertib) showed high levels of PLK1 protein resembling treatment‐naïve cell lines, suggesting the pivotal role in regulating the cell cycle. Hence, a thorough analysis is warranted to determine the exact role for tozasertib that targets polo‐like kinases and its implications on cellular resistance [47, 49].

### 3.6 Transcriptome profiling analysis reveals that PDK4 is involved in tozasertib resistance in malignant human glioma cell lines

**Fig. 6:**
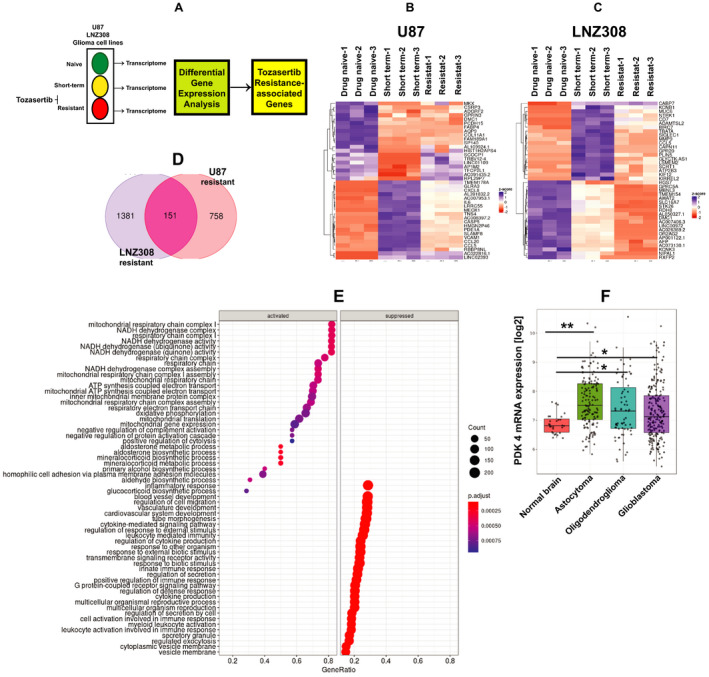
Transcriptome profiling analysis reveals that PDK4 is involved in tozasertib resistance in human malignant glioma cell lines. (A) Schematic of the approach taken to identify tozasertib‐responsive genes. (B, C) Heat map representation of the top 40 DEGs (based on log_2_ fold change and adjusted *P* value < 0.05) comparing drug‐naïve, tozasertib‐resistant (resistant), or cells treated with 100 nmol·L^−1^ tozasertib for 3 days (short‐term) in U87 cells (B), and LNZ308 cells (C). (D) Venn diagram indicating number of genes overlapping in U87‐resistant and LNZ308‐resistant cells between comparisons. (E) KEGG pathway enrichment plot comparing resistant to naïve LNZ308 cells. Each circle in the figure represents a pathway further split into activated and suppressed. The Y‐axis represents the name of the pathway, and the *X*‐axis indicates Enrichment Factor, indicating the proportion of the annotated genes to all genes in the pathway. (F) PDK4 mRNA expression across several glioma tumors compared with normal brain in the REMBRANDT dataset. Meta‐analysis performed in R2 genomics software comparing normal brain (cortex, *n* = 28), astrocytoma (*n* = 147), oligodendroglioma (*n* = 67), and GBM (*n* = 219) showed PDK4 mRNA expression was significantly upregulated compared to normal brain (**P < 0.001 normal brain vs astrocytoma; *P < 0.05 normal brain vs oligodendroglioma; *P < 0.01 normal brain vs GBM).

In order to identify gene expression signatures, and the molecules associated with acquired resistance, we isolated RNA from cells exposed to short‐term treatment with tozasertib and from a population of resistant cells (cultured in tozasertib for at least 8–10 weeks). RNA from drug‐naïve cells served as control (Fig. 6A). On average, 20 million sequencing reads were obtained for each sample (*N* = 3 biological replicates). Genes identified using DESeq2 that featured an adjusted *P* value of < 0.05, and log_2_ fold change of +1.5 were regarded as differentially expressed genes (DEGs). DEGs are displayed in Table S1. Hierarchical clustering of the top 40 DEGs markedly separated the control and various treatment groups (Fig. 6B,C). Interestingly, we found 151 upregulated genes common to both U87‐ and LNZ308‐resistant cells (Fig. 6D and Table S2).

The clusterProfiler package in R was utilized for the identification and visualization of enriched pathways among DEGs. Similar enrichment analysis was also conducted using GSEA. The dot plot depicts the significantly enriched KEGG pathways in tozasertib‐resistant cells when compared to the control cells. We then identified the emergence of a significant upregulation in pathways belonging to mitochondrial respiratory chain complex I, NADH dehydrogenase complex, NADH dehydrogenase activity, NADH dehydrogenase complex assembly, ATP synthesis coupled electron transport, oxidative phosphorylation, and mitochondrial gene expression in resistant cells (Fig. 6E, left half). In contrast, inflammatory response, vasculature development, tube morphogenesis, cytokine‐mediated signaling pathway, regulation of cytokine production, G protein‐coupled receptor signaling pathway, and cell activation involved in immune responses were suppressed in the resistant cells (Fig. 6E, right half). The most notable upregulated gene observed in tozasertib‐resistant cells was PDH kinase 4 (6.8‐ and 3.8‐fold‐increase in U87 and LNZ308, respectively) (Table S2), which is a member of a PDK family of isozymes (PDK1, PDK2, PDK3, and PDK4). To study the clinical relevance of PDK4 expression in tumors of central nervous system [50], we performed datamining studies using the REMBRANDT dataset. As shown in Fig. 6F, PDK4 mRNA expression was upregulated across several tumors including glioma when compared against normal brain in the REMBRANDT dataset.

### 3.7 Sustained exposure to tozasertib promotes PDK expression and inhibits PDH activity

**Fig. 7:**
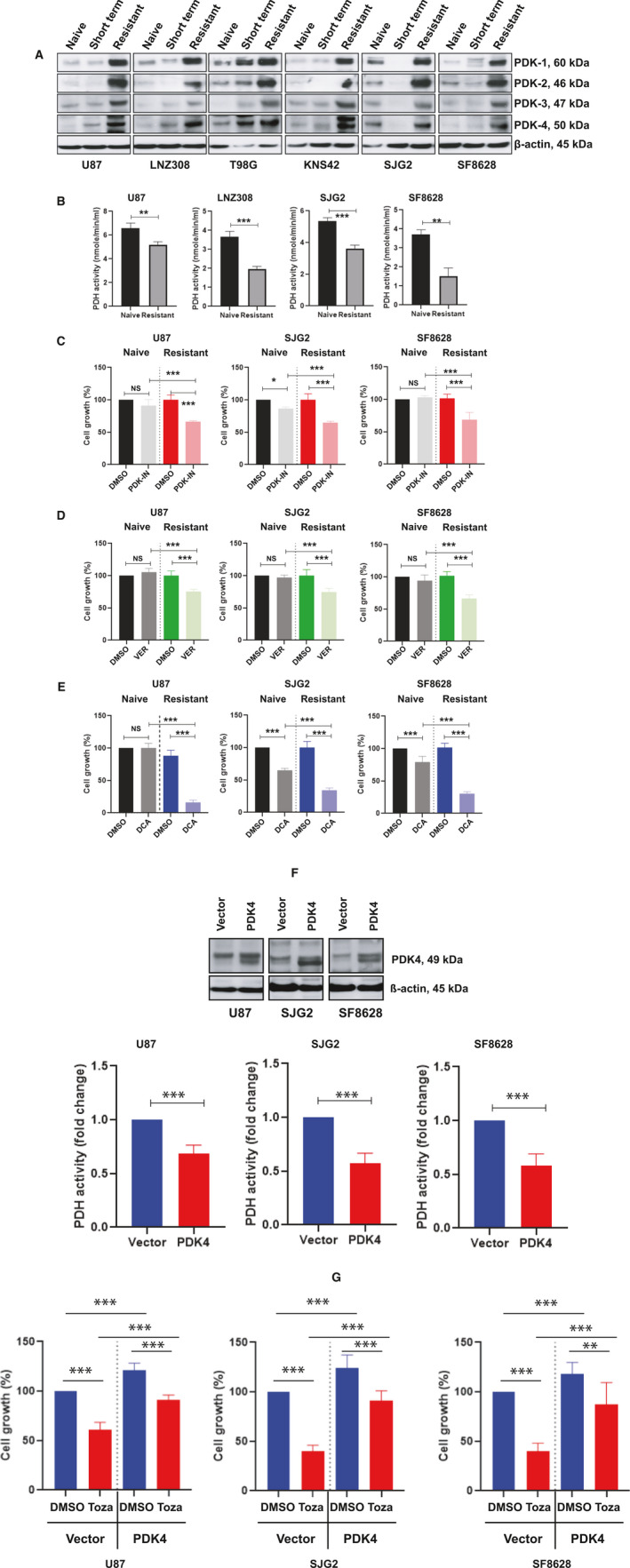
Sustained exposure to tozasertib promotes PDK expression and inhibits PDH activity. (A) Whole cell extracts from drug‐naïve, or cells treated with tozasertib for 72 h (short‐term), or tozasertib‐resistant cells were prepared. Twenty micrograms of protein were separated on a SDS/PAGE. Western blot analysis was performed with the indicated antibodies. Expression of β‐actin served as a loading control. These experiments were performed at least three times, and a representative blot is presented here. (B) Whole cell extracts from drug‐naïve or tozasertib‐resistant cells were subjected to PDH activity assay and reported as PDH activity·min^−1^·mL^−1^. The values are represented as mean ± standard deviation of three independent experiments (statistical analysis was carried out with an unpaired two‐tailed *t*‐test; **P < 0.005, ***P < 0.0001). (C–E) Drug‐naïve or tozasertib‐resistant adult (U87) and pediatric (SJG2 and SF8628) glioma cell lines were treated with 10 µmol·L^−1^ of PDK4‐IN‐1 hydrochloride (C) or 10 µmol·L^−1^ of VER‐246608 (D) or 1 mmol·L^−1^ DCA (E) for 72 h. Vehicle (DMSO) treated cells served as control. Seventy‐two hours later, cell growth was assessed by MTS assay. Absorbance was recorded at 490 nm using a Synergy HTX multimode microplate reader. The values are represented as mean ± standard deviation of three separate experiments (= four wells per condition). Data (C–E) were analyzed for statistical significance by Tukey's ANOVA (NS, not significant, *P < 0.01, ***P < 0.0005). (F, G) Logarithmically growing U87, SJG2, and SF8628 drug naïve or tozasertib‐resistant cells were transfected with empty vector or PDK4 expression plasmid. Forty‐eight hours after infection, cells were washed, lifted, and lysed. Equal amount of protein was separated on a SDS/PAGE and western blot analysis was performed with PDK4 antibody. Expression of β‐actin served as a loading control. These experiments were performed at least three times, and a representative blot (F, upper panel) is presented here. In parallel, cell lysates were analyzed for PDH activity and reported as fold change relative to control (F, lower panel). Data are the mean ± SD of three independent experiments (***P < 0.0005, data analyzed for statistical significance by unpaired two‐tailed *t*‐test). (G) Vector or PDK4‐transfected cells were treated with or without tozasertib (100 nmol·L^−1^) for 72 h. Vehicle (DMSO) treated cells served as control. Cell growth was assessed by spectrophotometric measurement of MTS bioreduction using a microplate reader (*n* = 4 wells per condition). Data point represents the mean ± SD of three measurements. Statistical analysis was carried out with Tukey's ANOVA (**P < 0.001, ***P < 0.0001).

Multiple studies have highlighted the role of the PDH pathway and of its control by PDKs in cancer cell metabolism [22, 51, 52]. Because PDKs phosphorylate and inactivate the PDH complex (PDHC), a process thought to promote cancer cell growth, we examined whether sustained exposure to tozasertib modulates the expression of these enzymes. As shown in Fig. 7A, under short‐term treatment conditions compared to vehicle‐treated control cells, glioma cells exhibited minimal or no change in PDK1, PDK2, PDK3, and PDK4 protein expression. However, tozasertib‐resistant cells exhibited a significant increase in PDK 1, 2, 3, and 4 protein levels. Because tozasertib‐resistant glioma cells increased PDK 1–4 protein levels, we then examined the activity of PDKs' regulatory target, PDH. Lysates from logarithmically growing glioma cells (naïve) and resistant cells were prepared and subjected to PDH activity analysis as measured by enzyme‐coupled colorimetric assay. As noted here, sustained treatment with tozasertib (i.e., resistant cells) significantly decreased PDH activity (Fig. 7B). Then, we selected PDK4‐IN‐1 hydrochloride, an anthraquinone derivative and a potent and orally active PDH kinase 4 inhibitor; VER‐246608, a potent and ATP‐competitive inhibitor of PDH kinases (PDK1, PDK2, PDK3, and PDK4); and DCA, a metabolic regulator in cancer cells' mitochondria with anticancer activity by inhibiting PDK activity; and examined the effect of these agents on cellular response. Drug‐naïve and resistant cell lines were treated with PDK inhibitors for 72 h and cell survival was assessed. We observed significant growth inhibition of tozasertib‐resistant cells compared to drug‐naïve cells exposed to PDK4‐IN‐1 hydrochloride (Fig. 7C; *P* < 0.0005 in U87, SJG2, and SF8628), VER‐246608 (Fig. 7D; *P* < 0.0001 in U87, SJG2, and SF8628), and DCA (Fig. 7E; *P* < 0.0001 in U87, SJG2, and SF8628). Together, these results demonstrated and validated the potency of PDK inhibitors to inhibit cell growth and survival of tozasertib‐resistant glioma cells. Although multiple studies reported the use of DCA (but not PDK4‐IN‐1 hydrochloride or VER‐246608) in humans, particularly in the treatment of cancer [53, 54], without affecting normal cells [55], we wanted to determine the vulnerability of human astrocytes. Non‐neoplastic human astrocytes were cultured in indicated concentrations of DCA and the loss of mitochondrial membrane potential elicited after 72 h was analyzed. Incubation of human astrocytes with DCA did not produce any noticeable change in mitochondrial membrane potential (Fig. S4).

To examine whether exogenous expression of PDK4 would reflect the resistant cell phenotype (on PDH activity and cell viability), logarithmically growing U87, SJG2, and SF8628 cells were transfected with PDK4 expression plasmids. Cells transfected with vector backbone served as control. Western blot analysis showed higher PDK4 expression than the vector‐transfected counterparts (Fig. 7F, upper panel). As shown in Fig. 7F lower panel, exogenous expression of PDK4 significantly reduced PDH activity and increased cell growth (Fig. 7G). In contrast, as shown in Fig. 7G, tozasertib inhibited cell growth (in the vector‐transfected cells) was significantly rescued by PDK4 overexpression, suggesting that PDK4 accumulation in glioma cells may promote cell survival and possibly during resistance development.

### 3.8 Increased mitochondrial biogenesis and mitochondrial membrane potential in tozasertib‐resistant cells is counteracted by PDH inhibition

**Fig. 8:**
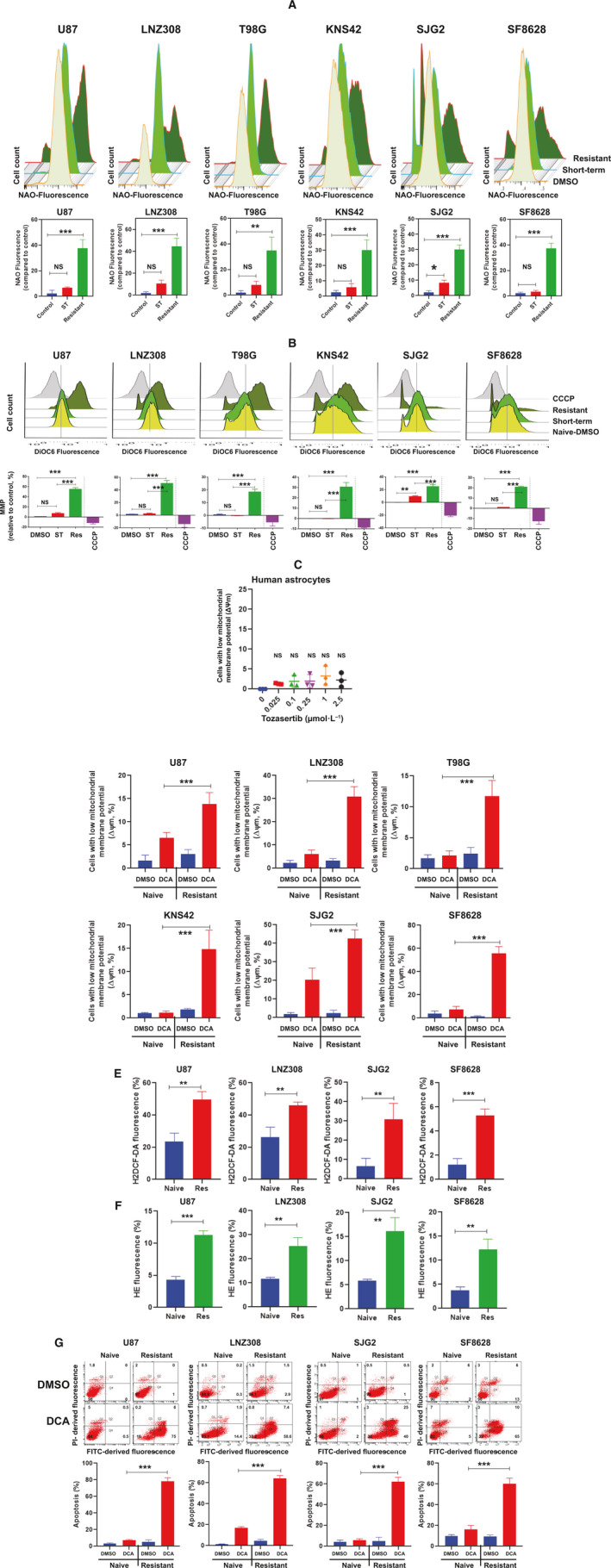
Increased mitochondrial biogenesis and mitochondrial membrane potential in tozasertib‐resistant cells is counteracted by PDH inhibition. (A) Drug naïve or tozasertib‐resistant (resistant) cells were allowed to attach overnight in a six‐well plate. Drug‐naïve cells were either treated with tozasertib for 72 h (short‐term) or vehicle (DMSO). Mitochondrial mass was measured by flow cytometric analysis after the cells were labeled with a fluorescent dye NAO. The representative FACS histogram is shown in the upper panel. The values are represented as mean ± standard deviation of three independent experiments. Data were analyzed by Tukey's ANOVA for statistical significance (NS, not significant; *P < 0.01; **P < 0.001; ***P < 0.0005). (B) Cells were subjected to mitochondrial membrane potential analysis by DiOC6 staining and flow cytometry. Drug‐naïve U87, LNZ308, T98G, KNS42, SJG2, and SF8628 or tozasertib‐resistant cells were treated with or without tozasertib (100 nmol·L^−1^) for 72 h. Vehicle (DMSO) treated cells served as control. Uncoupling of mitochondrial respiration with CCCP served as a positive control (note the appearance of a left‐shifted population of cells with reduced ∆ψm in CCCP‐treated cells, upper panel). Positively charged DiOC6 accumulates more in intact mitochondria, whereas depolarized mitochondrial membranes accumulate less DiOC6. A representative FACS plot and the data obtained from multiple experiments demonstrated loss of mitochondrial membrane potential (upper panel). The loss of mitochondrial membrane potential (left‐shifted population) or hyperpolarized mitochondrial membrane potential (right‐shifted population) was quantified. Results from the mean ± standard deviation of three separate experiments are shown in the lower panel. Data were analyzed by Tukey's ANOVA (NS, not significant, ***P < 0.0005). (C) Human astrocytes were subjected to mitochondrial membrane potential analysis by DiOC6 staining and flow cytometry. The change in mitochondrial membrane potential (compared to DMSO‐treated control, 0) was measured. These experiments were performed at least three times, and the values are represented as mean ± standard deviation. Results were analyzed by Tukey's ANOVA for statistical significance (NS, not significant). (D) Drug‐naïve U87, LNZ308, T98G, KNS42, SJG2, and SF8628 or tozasertib‐resistant cells were treated with or without DCA (1 mmol·L^−1^) for 72 h. Control cells received DMSO. Cells were subjected to mitochondrial membrane potential analysis by DiOC6 staining and flow cytometry. The loss of mitochondrial membrane potential was quantified. These experiments were performed at least three times, and the values are represented as mean ± standard deviation. Treatment of tozasertib‐resistant cells significantly increased the cell population with low ∆ψm (***P < 0.0001, analysis was carried out with Tukey's ANOVA). In contrast, we noticed a minimal or modest change in the intrinsic cellular mitochondrial membrane potential in the naïve cells cultured in the presence of DCA for 72 h. (E, F) Drug‐naïve U87, LNZ308, SJG2, and SF8628 or tozasertib‐resistant cells were treated with DCA for 72 h, and then, the cells were labeled with H2DCFDA and hydroethidine to analyze hydrogen peroxide (E) and superoxide anion (F), respectively, by flow cytometry. These experiments were performed at least three times, and the values are represented as mean ± standard deviation (**P < 0.01, ***P < 0.005; unpaired two‐tailed *t*‐test was used to assess the statistical significance). (G) Drug naïve U87, LNZ308, SJG2, and SF8628 or tozasertib‐resistant cells were treated with or without DCA (10 mmol·L^−1^) for 72 h. Control cells received DMSO. Cells were subjected to apoptosis analysis by flow cytometry. When tozasertib‐resistant cells were treated with DCA, a large population of cells accumulated in the Q4 and Q2 quadrants, suggesting that the cells were undergoing early and late phase of apoptosis/necrosis. These experiments were performed at least three times, and the values are represented as mean ± standard deviation. Tukey's ANOVA was used to assess the statistical significance (***P < 0.0001, lower panel).

Given the role of PDKs in mitochondrial biogenesis and regulation of mitochondrial energy production pathways [56, 57, 58, 59], we then examined the effect of tozasertib on the mitochondrial biogenesis and mitochondrial membrane potential. We used 10‐*N*‐nonyl acridine orange (NAO), a metachromatic dye, which binds to the mitochondrial‐specific phospholipid, cardiolipin, where increased fluorescence intensity signal correlates well with increased mitochondrial content [60, 61]. As shown in Fig. 8A, minimal or modest change (mitochondrial biogenesis) was observed after exposure to 100 nmol·L^−1^ tozasertib for 72 h. However, we noticed a significant increase in the relative intensity of the fluorescent dye (Fig. 8A, upper panel) when the cells were subjected to sustained tozasertib treatment (tozasertib‐resistant cells). Compared to drug‐naïve control, the resistant cell population exhibited high NAO intensity in both adult (37%, 44%, and 35%, U87, LNZ308, and T98G, respectively), and pediatric (30%, 30%, and 37%, KNS42, SJG2, and SF8628, respectively) cells (Fig. 8A, lower panel), suggesting that sustained exposure to tozasertib may be mediating the increased mitochondrial biogenesis. Similarly, the mitochondrial membrane potential, a critical bioenergetic parameter controlling mitochondrial ROS production, was examined using the cationic dye, DiOC6 [62]. DiOC6 fluorescence was abolished by the uncoupling agent carbonyl cyanide m‐chlorophenyl hydrazone (CCCP), which served as positive control (note the left shift due to loss of mitochondrial membrane potential). As shown in the Fig. 8B (upper panel), the intensity of the fluorescence staining was indistinguishable between drug‐naïve control and short‐term tozasertib‐treated cells. In contrast, sustained exposure to tozasertib led to 55%, 50%, 18%, 30%, 24%, and 20% increase in U87, LNZ308, T98G KNS42, SJG2, and SF8628, respectively (Fig. 8B, lower panel), suggesting that the redox‐regulated mechanisms of mitochondrial biogenesis and increased mitochondrial membrane potential could enhance the capacity to support the cell's metabolic needs during the periods of oxidative stress. Of note, we conducted experiments to examine the effects of tozasertib on δψm in non‐neoplastic human astrocytes. As shown in Fig. 8C, as high as 2.5 µmol·L^−1^ tozasertib produced no change in δΨm, suggesting that even quite high concentrations of tozasertib are apparently non‐toxic, at least during the timeframe of our experiments.

Finally, because there is a strong association between PDK overexpression and drug resistance [63], we hypothesized that DCA might help to sensitize tozasertib‐resistant cells. Incubation of cells with DCA for 72 h shows a significant increase in the low mitochondrial membrane potential in the tozasertib‐resistant cell population (Fig. 8D). Furthermore, DCA significantly increased oxidative injury (Fig. 8E,F) and cell death (Fig. 8G), suggesting that PDKs could be a promising therapeutic target for tozasertib‐induced resistance in glioma.

## 4 Discussion

The novel mechanism of action, tolerability, and oral formulation make aurora kinase inhibitors a promising class of agents that appears to have activity in both hematological malignancies and solid tumor types [64, 65, 66]. However, the positive response often occurred after multiple rounds of treatment [67, 68], as compared to rapid anti‐tumor effects observed in preclinical studies [69, 70], suggesting that longer treatment durations may be necessary before measurable disease stabilization [71]. Although many novel small molecules that target aurora kinases are now in clinical trials [47, 72, 73, 74], it appears that even those tumors that respond initially exhibit a tendency to acquire resistance and recur. In this study, we showed that the aurora kinase inhibitor tozasertib was effective in killing glioma cells while sparing non‐neoplastic human astrocytes. To substantiate this notion, we carried out annexin V/PI binding assays, which showed that tozasertib increased the percentage of annexin V positive and PI positive apoptotic cells. Further studies on cell cycle analysis revealed that tozasertib treatment increased the sub‐G1 population, which represent cells with significant DNA damage indicating a late apoptotic stage with respect to cycling cells. Further, tozasertib treatment suppresses cell proliferation and migratory ability in glioma cell lines. Cell cycle analysis also demonstrated that aurora kinase inhibition produced a significant increase in cells with 4N and higher DNA content, consistent with the mechanism of these agents in inducing mitotic arrest, which also translated into an increase in cellular senescence. Moreover, tozasertib induced generation of ROS and mitochondrial dysfunction, counteracting one of the carcinogenic mechanisms of aurora kinases in antagonizing ROS production [3, 9, 41, 42], which was blocked by NAC.

However, prolonged treatment with tozasertib led to the emergence of a resistant population that maintained normal proliferation despite continued tozasertib treatment, a phenomenon observed in all six glioma cell lines (adult, U87, LNZ308, and T98G; pediatric, KNS42, and SJG2 and pediatric brain stem glioma, SF8628). We employed several strategies to elucidate the molecular basis for acquired resistance in these glioma models. Based on western blotting analysis, we observed aurora kinase A, B, and C protein expression was significantly upregulated in all resistant cell lines. In addition, cell cycle profile in resistant cells was similar to their drug‐naïve counterparts, and resistant cells proliferated at a similar rate as parental cells, suggesting that these cells had become refractory to clinically achievable concentrations of tozasertib. Importantly, the emergence of resistance to tozasertib was not associated with the p53 status of the tumor cell lines (e.g., U87 was p53 wild‐type, whereas LNZ308 was p53 deleted) and cell cycle analysis showed a paucity of cells with 4N or greater DNA content, a sharp contrast to short‐term treated cells. Because studies of drug‐induced resistance in cell line models can suggest strategies to circumvent drug resistance in the clinical setting, we examined the effect of alisertib (MLN8237, Millennium Pharmaceuticals, Cambridge, MA, USA), barasertib (AstraZeneca, previously known as AZD1152‐hydroxyquinazoline pyrazol anilide, HQPA), and ZM447439 (AstraZeneca). Under *in vitro* conditions, alisertib, barasertib, and ZM447439 displayed broad anticancer activity in logarithmically growing treatment‐naive glioma cells. Although aurora kinase mutations and hyperexpression have proven to be relevant resistance mechanisms in other tumor types [49, 75, 76], the aurora kinase overexpression in our treatment‐resistant glioma cell lines did not appear to be the fundamental mechanism of resistance in view of our observation that treatment with increasing concentrations of alisertib, barasertib or ZM443479 produced no significant decrease in cell growth. These observations did not definitively rule out a resistance‐inducing mutation, but the uniformity of the resistance induction across cell lines prompted us to explore the possibility of a more global response–escape mechanism.

In this context, we noticed an extensive change in gene expression that accompanied tozasertib treatment. Between U87 and LNZ308 tozasertib‐resistant cell lines, 151 genes overlapped. Tozasertib‐resistant glioma cells significantly upregulated the expression of PDH kinase 4, a negative regulator of glucose oxidation, as assessed using RNA‐seq analysis, in comparison with drug‐naïve controls. Western blot analysis revealed that all four PDK isoenzymes (PDK1, PDK2, PDK3, and PDK4) were upregulated in resistant cell lines. Moreover, the TCGA database showed that PDK4 mRNA levels were upregulated and are correlated with poor survival of GBM [50] and other cancer patients [77, 78, 79]. PDK is a mitochondrial enzyme that is overexpressed in different cancers and results in the selective inhibition of PDH that converts cytosolic pyruvate to mitochondrial acetyl‐CoA, the substrate for the tricarboxylic acid (TCA) cycle. Several recent studies have specifically implicated overexpression of aurora kinase in enhanced mitochondrial function, drug resistance, and tumor cell invasion [45, 80, 81, 82]. Recently, Hsieh *et al*. [83] identified 150 differentially expressed proteins after tozasertib treatment in the Th‐MYCN mouse model. Their network‐based enrichment analysis revealed that tozasertib alters metabolic processes particularly in fatty acid β‐oxidation. In a similar fashion, we showed a significant increase in the mitochondrial biogenesis in tozasertib‐resistant cells which may be crucial for controlling ROS‐mediated DNA damage. These results suggest that the increased mitochondrial biogenesis and respiratory capacity can support increased proliferation and metabolic activity associated with resistant cell growth [84, 85, 86].

In this report, we found the levels of PDK1, PDK2, PDK3, and PDK4 protein expression increased in the tozasertib‐resistant cells, with resultant inactivation of PDH activity. In accordance with Woolbright *et al*. [79] in bladder cancer chemoresistance, we demonstrated that ectopic expression of PDK4 in glioma cells results in a significant increase in cell growth, which is consistent with the observation that higher PDK4 expression is associated with a poor prognosis in patients with glioma and with other solid tumors [50, 77, 78, 79].

Although numerous mechanisms are responsible for cancer resistance to chemotherapy, an emerging concept suggests that metabolic change in cancer cells may be a critical determinant of resistance that is due, in part, to an attenuated mitochondrial function, which results from the inhibition of PDHC via increased expression of PDK1, PDK2, PDK3, and PDK4 [87, 88]. Several studies have reported PDKs as potential molecular targets for cancer and other metabolic diseases [78, 89, 90, 91, 92, 93, 94, 95]. PDK4 has been shown to interact directly with E2F1 and influences mitochondrial glucose oxidation and cellular bioenergetics [96]. Inactivation of PDKs using inhibitors such as PDK4‐IN‐1 hydrochloride (an anthraquinone derivative and a potent and orally active PDK4 inhibitor), VER‐246608 (a potent and ATP‐competitive inhibitor of PDK with IC50s in the low nanomolar range for PDK1, PDK2, PDK3, and PDK4) and the pyruvate analog DCA (the most common classic inhibitor of PDK isoforms, including PDK4) displayed a higher degree of efficacy in tozasertib‐resistant cells. This was in agreement with the previously published reports that inactivation of PDKs in various cancer cell lines, either by the small molecule inhibitor DCA [55] or molecular (siRNA) approaches [97], leads to increased activity of PDH, thereby changing cellular bioenergetics and restoring mitochondria‐dependent apoptosis [98, 99]. In agreement with this hypothesis, we observed that treatment of tozasertib‐resistant cells with DCA (used because direct preclinical evidence of anticancer effects has been published with glioma, breast, non‐small cell lung cancer, prostate, and endometrial cancer [53, 54]) reduced cell viability and increased loss of mitochondrial membrane potential, an effect not observed in the drug‐naïve controls and normal non‐neoplastic human astrocytes. These observations suggest that DCA, by inhibiting PDK and hence activating PDH, promotes mitochondrial depolarization and mitochondrially mediated cell death [23, 100, 101], as a selective vulnerability in tozasertib‐resistant cells.

## 5 Conclusions

The results suggest that clinically relevant concentrations of tozasertib, a pan‐aurora kinase inhibitor, has significant pro‐apoptotic activity in adult and pediatric glioma cell lines (compared to little or no effect on normal human astrocytes). However, the challenge presented is neoplastic cells acquire resistance at clinically achievable dosages, limiting the effectiveness of this therapeutic agent. We demonstrated that, over time, the emergence of this resistance to tozasertib is generated due to the neoplastic cells' highly integrated metabolic PDK/PDH network, driving mitochondrial biogenesis, and promoting this resistant cell growth. This convergent mechanism of resistance offers potential therapeutic opportunities to re‐sensitize the cells to tozasertib.

## Conflict of interest

The authors declare no conflict of interest.

## Author contributions

EPJ and DRP contributed equally to this work. IFP and DRP conceived and designed the study. EPJ, DRP, SA, and DR involved in development of methodology. EPJ, DRP, DR, MCR, and ST acquired data (provided animals, acquired, and managed patients, provided facilities, etc.). EPJ, DRP, and DR analyzed and interpreted the data (e.g., statistical analysis, biostatistics, computational analysis). Writing, review, and/or revision of the manuscript: DPR, DR and IFP. All the authors edited and approved the manuscript.

### Peer Review

The peer review history for this article is available at https://publons.com/publon/10.1002/1878‐0261.13025.

## Supporting information


**Fig. S1**. Cell cycle profiles in response to the inhibition of aurora kinases in malignant human glioma cells. (A, B) Logarithmically growing adult (U87, LNZ308 and T98G) and pediatric (KNS42, SJG2 and SF8628) glioma cells were seeded on 6‐well plates (1 × 10^5^). On the following day cells were either transfected with non‐target siRNA (NT siRNA) or aurora kinase A or B or C siRNA. Seventy‐two hours post‐transfection, the cells were trypsinized, lifted, washed, and stained with PI. Fluorescence of the PI‐stained cells was measured using a Becton Dickinson flow cytometer. The color‐coded bar graphs show the percentage of glioma cells in various phase of cell cycle such as sub‐G1, G1, S, G2M and > 4N DNA content (A). B represents the average percentage of cells accumulated from three different experiments. (C) Logarithmically growing adult (U87, LNZ308 and T98G) and pediatric (KNS42, SJG2 and SF8628) cells were seeded on 6‐well plates (1 × 10^5^). After overnight attachment period, cells were treated with tozasertib for 24 h or 72 h. After treatment, the cells were washed, trypsinized, and lifted. Then the cells were stained with PI and the fluorescence of the PI‐stained cells was measured using a flow cytometer. Quantitative measurements of cell cycle profile were performed, and the percentage of cells accumulated in different phases of the cell cycle from three different experiments are shown.

**Fig. S2**. Schematic of the approach taken to achieve drug resistance. Malignant human glioma cells were exposed to increasing concentrations of tozasertib. Starting with an initially low dose (10 nmol·L^−1^), the inhibitor concentration was progressively increased to 100 nmol·L^−1^ over a period of several weeks.

**Fig. S3**. Cross‐resistance to other aurora kinase inhibitors. (A) Drug‐naïve or tozasertib‐resistant (resistant) cells were allowed to attach overnight in a 6‐well plate. Drug‐naïve cells were either treated with tozasertib for 72 h (short‐term) or an equal amount of vehicle (DMSO). After treatment, the cells were trypsinized, lifted, washed, and stained with PI. Fluorescence of the PI‐stained cells was measured using a Becton Dickinson flow cytometer and the percentage of cells accumulated in different phases of cell cycle from three different experiments are shown. (B) Logarithmically growing drug‐naïve or tozasertib‐resistant cells (3 × 10^3^) were seeded on 96‐well plates. After overnight attachment period, cells were exposed to indicated concentrations of inhibitors (alisertib, left panel; barasertib, middle panel and ZM447439, right panel) for 72 h. DMSO (0) served as vehicle control. Cell growth was assessed spectrophotometric measurement of MTS bioreduction. Absorbance was recorded at 490 nm using a Synergy HTX multimode microplate reader. The values are represented as mean ± standard deviation of three separate experiments. (C) Whole cell extracts from drug‐naïve or cells treated with tozasertib for 72 h (short‐term) or tozasertib‐resistant cells were prepared. Twenty micrograms of protein were separated on a SDS/PAGE. Western blot analysis was performed with the polo‐like kinase 1 (PLK1) antibody. Expression of β‐actin served as loading control. These experiments were performed at least three times, and a representative blot is presented here.

**Fig. S4**. The effect of DCA on mitochondrial membrane potential of normal human astrocytes. Non‐neoplastic human astrocytes were treated with the indicated concentrations of DCA for 72 h. Then, the cells were subjected to mitochondrial membrane potential analysis by DiOC6 staining and flow cytometry. No significant change in the intrinsic cellular mitochondrial membrane potential was evident in the cells cultured in the presence of 2.5 mmol·L^−1^ DCA for 72 h. The values are represented as mean ± standard deviation of three independent experiments (NS, not significant, Tukey's ANOVA was used to examine the statistical significance).

**Table S1**. List of up‐ and down‐ regulated DEG for the LNZ308 and U87 samples at a 5% threshold, and log‐fold change of 1.5.

**Table S2**. List of common genes between LNZ308 and U87 samples.

## Data Availability

No datasets were generated or submitted related to this paper that are available in a public database.
